# First-Principles Investigations of Novel Guanidine-Based Dyes

**DOI:** 10.1021/acsomega.3c09182

**Published:** 2024-03-14

**Authors:** Uzma Hashmat, Nasir Rasool, Samia Kausar, Ataf Ali Altaf, Sabiha Sultana, Asif Ali Tahir

**Affiliations:** † Department of Chemistry, Government College University, Faisalabad 38000, Pakistan; ‡ Environment and Sustainability Institute (ESI), Faculty of Environment, Science and Economy, University of Exeter, Penryn Campus, TR10 9FE Cornwall, U.K.; § Department of Chemistry, University of Gujrat, Hafiz Hayat Campus, Gujrat 50700, Pakistan; ∥ Department of Chemistry, University of Okara, Okara 56300, Pakistan; ⊥ Department of Food Science, College of Agriculture and Life Sciences, Cornell University, Ithaca,New York 14853, United States; # Department of Chemistry, Islamia College University, Peshawar 25120, Pakistan

## Abstract

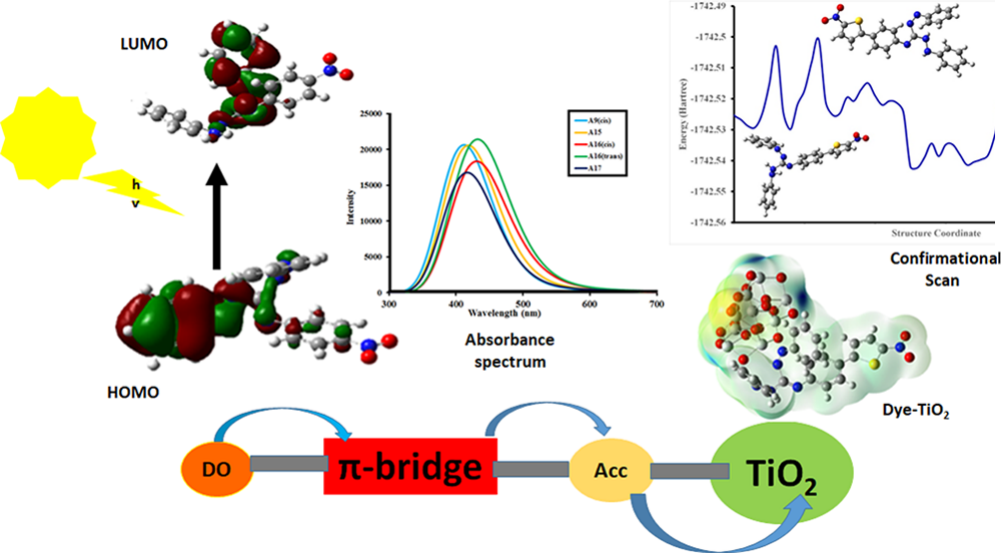

In the pursuit of finding efficient D-π-A organic dyes as photosensitizers for dye-sensitized solar cells (DSSCs), first-principles calculations of guanidine-based dyes [**A1**–**A18**] were executed using density functional theory (DFT). The various electronic and optical properties of guanidine-based organic dyes with different D-π-A structural modifications were investigated. The structural modification of guanidine-based dyes largely affects the properties of molecules, such as excitation energies, the oscillator strength dipole moment, the transition dipole moment, and light-harvesting efficiencies. The energy gap between the highest occupied molecular orbital (HOMO) and the lowest unoccupied molecular orbital (LUMO) is responsible for the reduction and injection of electrons. Modification of the guanidine subunit by different structural modifications gave a range of HOMO–LUMO energy gaps. Chemical and optical characteristics of the dyes indicated prominent charge transfer and light-harvesting efficiencies. The wide electronic absorption spectra of these guanidine-based dyes computed by TD-DFT-B3LYP with 6-31G, 6-311G, and cc-PVDZ basis sets have been observed in the visible region of spectra due to the presence of chromophore groups of dye molecules. Better anchorage of dyes to the surface of TiO_2_ semiconductors helps in charge-transfer phenomena, and the results suggested that −COOH, −CN, and −NO_2_ proved to be proficient anchoring groups, making dyes very encouraging candidates for DSSCs. Molecular electrostatic potential explained the electrostatic potential of organic dyes, and IR spectrum and conformational analyses ensured the suitability of organic dyes for the fabrication of DSSCs.

## 1 Introduction

Solar energy is recently considered a great source of energy that fulfills the demand for electricity due to its low-cost production as conventional or nonrenewable energy sources are being depleted with the increase in population. The solar cells function effectively by producing environmentally friendly gases with no toxic products or noise. Dye-sensitized solar cells (DSSCs) are considered to have great potential due to their low fabrication cost and high efficiency in electricity production.^1−5^

**Figure 1:**
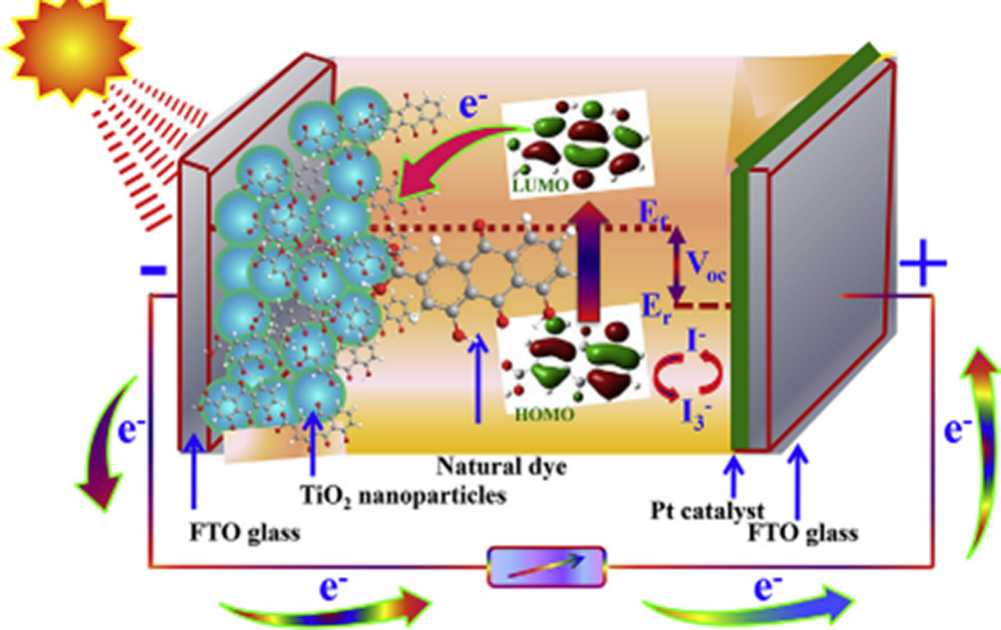
Working scheme of DSSCs from ref (8) reused with permission from Elsevier license number 5712010860265.

DSSC is composed of many components, such as semiconductors, dye sensitizers, redox electrolytes, and counter electrodes. Solar light is used as a source for the excitation of electrons in organic dyes from their ground state of energy. DSSC has a sandwich-like structure containing two TCO substrates. TCO has a temperature resistance property that is suitable for the TiO_2_ layer and the transmittance of light. The excited electrons of the oxidized dye are then inserted into the conduction band (CB) of a wide band gap semiconductor (usually TiO_2_).^6^ From the CB, electrons are transferred to the transparent conducting glass. These electrons are transported to the electrolyte on the counter electrode by employing an external circuit. Dye gains the electrons from the electrolyte in the dye regeneration process, turning the electrolyte molecules electron-deficient, which diffuse toward the counter electrode.^7^ The restoration of the initial state of the electrolyte happens on the counter electrode due to the reduction process taking place through a redox mediator. The working process of the DSSCs is shown in Figure 1.

The DSSC performance depends on the photosensitive dye bonded on TiO_2_, which remarkably affects its efficiency.^9^ A photosensitized dye must have a broad spectrum with a wide absorption. LUMO is confined to the acceptor unit. More electronegativity of the acceptor component of the dye localized the LUMO on the acceptor.^10^ To avoid carrier recombination processes, HOMO should be located farther from the TiO_2_ semiconductor. For efficient electron injection, the energy gap of HOMO–LUMO levels of excited dyes must be higher than the CB of semiconductor TiO_2_ in terms of electronic structure.^11^ The value of the HOMO of dye molecules must be lesser than the HOMO of the electrolyte (I^–^/I_3_^–^) redox couple of the dye molecule.^12^ In another step, the photosensitizing process in which the excited electron is directly transferred from the dye at its ground state to the wide CB of the semiconductor is gaining very much importance and recognition due to its more efficient procedure. Furthermore, maximum absorption in the visible region with a wide range of wavelength is responsible to calculate the light-harvesting efficiency (LHE).^13−15^

Photosensitized dyes must be stable in their excited state as well as in their ionized state to increase their efficiency and consistency, which can be evaluated through an analysis of their chemical reactivity parameters. The molecular structure in good flexibility is fundamentally very important.^3^ The efficiency and stability of organic dyes are key aspects in improving the performance of DSSCs. Organic dyes in DSSCs consist of a donor (D) moiety linked with an acceptor moiety (A) through π-conjugation as a D-π-A structure, which is responsible for developing a variety of organic dyes.^16^ They have a basic D-π linker-A structure having π-bridged dye molecules, which link the donor part with the acceptor part of dye molecules. The acceptor unit of dye molecules is responsible to confine the molecule on the semiconductor surface, while the π bridging molecule, due to its conjugation, is responsible for easy charge transfer and efficient light harvesting over a wide range.^17−20^

Large organic molecules are not easily synthesized by simple processes, so before their preparation in the lab, it is necessary to predict their LHE computationally.^21^ Various efforts have been made for the theoretical investigation of characteristics of dyes, which explain the modified design of new dyes.^22−27^ Since the process and function of DSSCs are based on excitation and transport of electrons, quantum-based calculations are used as a guiding principle for the molecular design of dyes.^28^ Density functional theory (DFT) calculations play an effective role in predicting the geometry of dyes at the ground state.^29^ To understand the electronic structure of dye molecules, Kohn–Sham (KS) molecular orbital analysis play a fundamental role in understanding the electronic structure of dyes. Time-dependent density functional theory (TD-DFT) methods are used to evaluate the excited states and the optical and electronic properties of dyes.^30^

In this work, we focus on the study of D-π-A structures containing variable donor and acceptor moieties. The π-linkages contain phenyl, thiophene, imine, and phenyl groups. It has been considered that these groups play a significant role in lowering the HOMO–LUMO gaps and covering a long-range of visible spectra. Literature studies indicate that dyes with a longer range of visible absorption spectrum have greater potential as light-harvesting agents for DSSCs.^31^ The objective of our work is to design modified dyes by using variable donor and acceptor moieties on the primary molecular structure of guanidine and finding the dyes that can be employed in DSSCs with efficient characteristics analyzed through quantum calculations.

## 2 Computational Details

### 2.1 Methods

To study the charge density, electronic transition, and absorption spectra of molecules, TD-DFT is being used.^32^ TD-DFT is used to efficiently study the oscillatory strengths of transitions and their excitation energies. For TD-DFT calculations, time-dependent perturbation (TD perturbation) was applied to the ground state of the molecule.^33^ TD-DFT also provides calculations about the charge density response, which in turn determines the excitation spectrum in the dipole approximation. Frequency-dependent polarizability α̅(ω)^34^ is the study of dipole moment (DM) to oscillation in the TD-DFT field and given below as eq 1
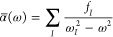
1where ω_*I*_ = excitation energy and *f*_*I*_ = oscillator strength. Frequency-dependent local density approximation (LDA) is employed by Gaussian 16 which provides potential change due to perturbation in the applied electric field. Fractional derivative^35^ provides kernel *f*_xc_ as under (eq 2):
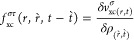
2where *v*_xc_^σ^ = exchange correlation for σ spin density of electron and ρ_τ_ = density of τ-spin of electron.^36^ In order to calculate the oscillator strength and energies of excitation, the eigen value equation ω_*i*_ is given as under (eq 3):
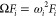
3where Ω = index matrix related to spin and *F*_*i*_ = eigen vectors. DFT is used to study the optical and electronic behaviors of observed molecules by operating the KS framework. These DFT calculations about energy states helped to study various optical properties such as the ionization energy, global hardness (η), chemical potential (μ), electron affinity, transfer of charge, and DM of molecules. The DFT gives the total energy of molecules as *E*(ρ) = *E*_e_(ρ) + ∫ *V*(*r*)ρ(*r*)*dr* where *E*_e_ is the electronic energy, *V* stands for the potential to calculate the attraction between the nucleus and the electron, and ρ is the electron density.^37^

### 2.2 Calculations

DFT and TD-DFT were carried out computationally in Gaussian 16 to calculate the ground-state geometries of 20 guanine-derived dye molecules (**A1**–**A18)** which were optimized under B3LYP functional with 6-31G, 6-311G, and cc-PVDZ basis sets. BPV86, LSDA, and B3PW91 functionals were also utilized for some compounds to study the optical and electric parameters. The DFT calculations were carried out in Gaussian 16W by using B3LYP along with the LanL2DZ basis set to study the interaction of the TiO_2_ semiconductor with the organic dye. The chemical structures of dye molecules (**A1**–**A18)** are given in Table 1.

**Table 1:**
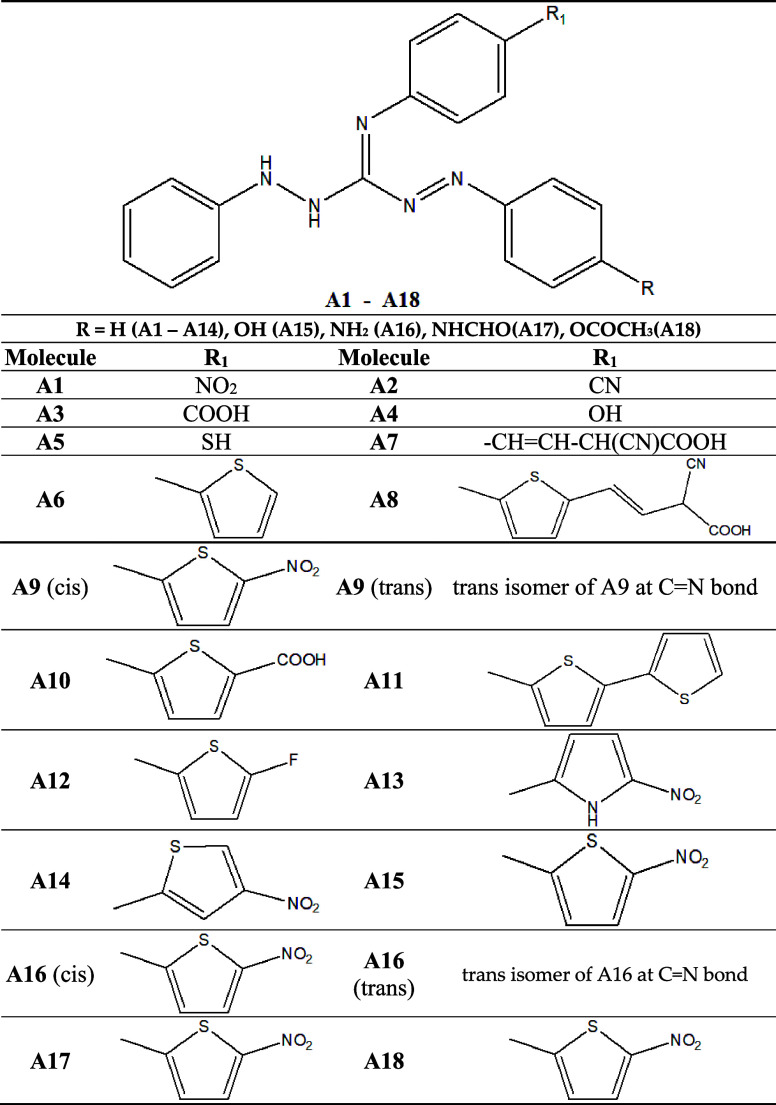
Chemical Structures of Guanine-Derived Dye Molecules (**A1**–**A18**)

HOMO is related to the donor moiety and LUMO is related to the electron acceptor unit of a molecule. The HOMO–LUMO energy gap was used to explain the electronic and optical properties of molecules, such as the chemical reactivity and stability of dye molecules.^38^ KS framework was able to calculate the electronegativity (*x*), chemical hardness (η), and chemical potential (μ) by using the following eqs 4, 5, and 6. IP (−*E*_HOMO_) stands for the ionization potential of the HOMO, and EA (−*E*_LUMO_) is related to the electron affinity of the LUMO. The global softness (σ) of dye molecules can be calculated by using following eq 7.^39^ Parr et al.^40^ found the electrophilicity index (ω) that is used to calculate the strength of electrophilicity using eq 8.^41^
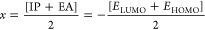
4
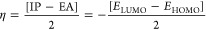
5
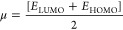
6
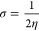
7
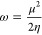
8

## 3 Results and Discussion

The electronic structures of all guanidine-based organic dyes with different D-π-A structural modifications are geometrically optimized, as given in Table 1. We attempted to get the values of desired properties of dyes with D-π-A structures through structural modification, which is helpful in calculating the energy levels of frontier molecular orbitals (FMOs). The guanidine central unit is substituted with some electron donor moieties, e.g., NH_2_ in some structures acts as the donor (D) part, which is linked with the π-linker phenyl ring. Thiophene substituted with different functional groups, i.e., −COOH, – CN, and −NO_2_, acts as an acceptor (A) moiety. These functional groups proved to be proficient anchoring groups on the surface of semiconductor TiO_2_, which supports efficient charge transfer and makes them very effective candidates to enhance the efficiency of DSSCs.

### 3.1 Molecular Orbital Analysis

**Figure 2:**
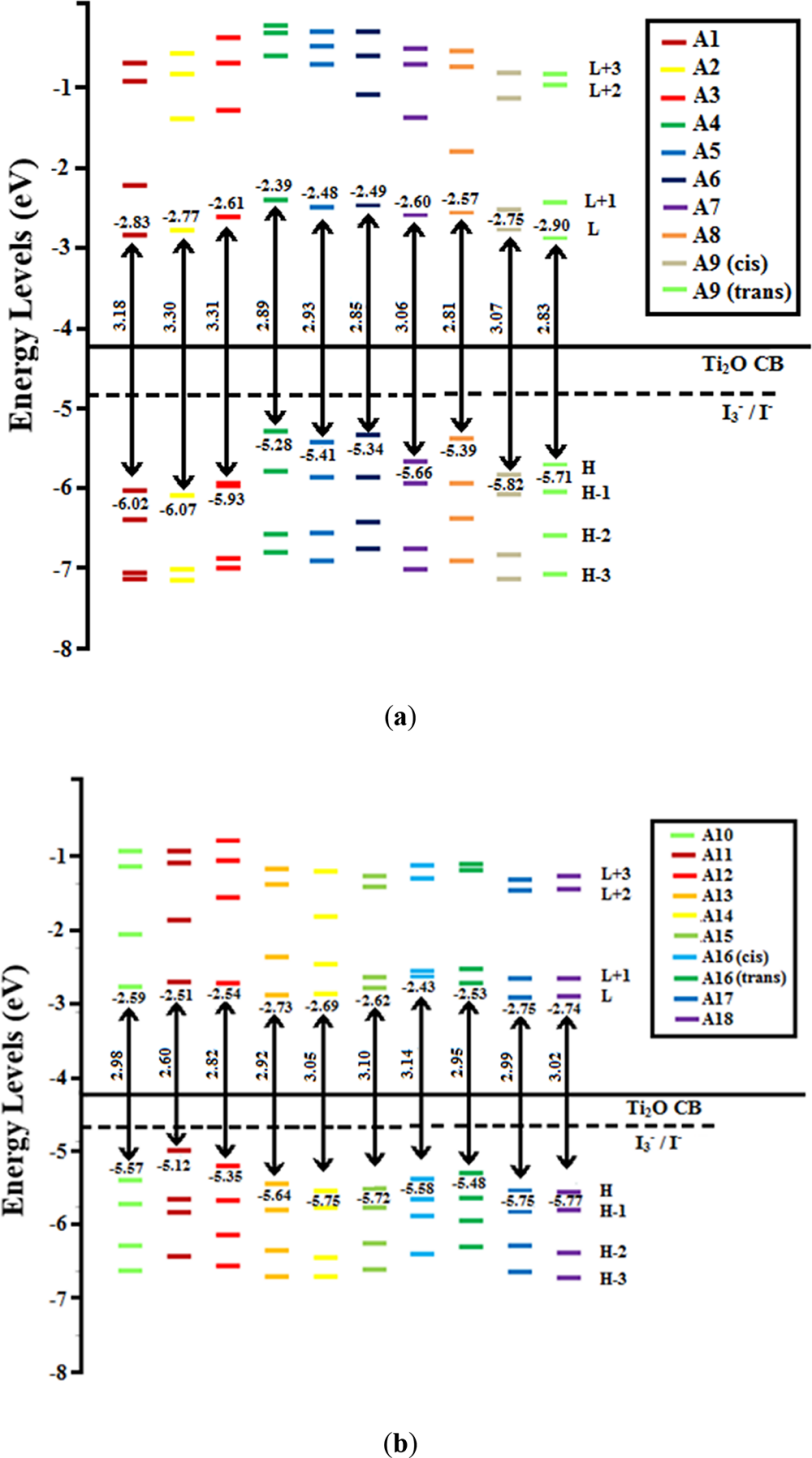
(a) Schematic molecular orbital energy levels of guanidine-based dyes [**A1**–**A9(trans)**]and CB of TiO_2_ electrolyte used as the reference. (b) Schematic molecular orbital energy levels of guanidine-based dyes (**A10**–**A18**)and CB of TiO_2_ electrolyte used as the reference.

In the FMO analysis of dye molecules, the HOMO is associated with the donor part of the molecule, and the LUMO is related to the acceptor unit of the molecule. Introducing a variety of functional groups in guanidine nuclei may alter the HOMO–LUMO gap, which obviously alters the charge transfer. This alteration in the HOMO–LUMO energy gap is very effective for studying the electronic and optical properties of dye molecules. The concentration of electron density of HOMO-3 is higher than the concentration of electron density in HOMO, HOMO-1, and HOMO-2. The contribution of HOMO-3 is greater from the nitrogen atom of the basic unit of guanidine and conjugated linked units. The major contribution of almost more than 40% of LUMOs of dye molecules, which are limited on the acceptor part, is from the central part of guanidine. Some contribution of LUMOs is due to the presence of specific functional groups attached to thiophene and benzene rings. The charge density of LUMO among LUMO-1, LUMO-2, and LUMO-3 is greater for all dyes. HOMO levels of all dyes are found to be close to each other in contrast to LUMOs with a remarkable difference in energy, as given in Figure 2a,b.

The changes in the positions of HOMOs, LUMOs, and the HOMO–LUMO gap are due to the structural modifications of dye molecules. Such changes in FMOs describe the transition of charge transfer due to the structural modification of molecules. The energy gap between HOMO and LUMO explains the stability of the molecule and other optical parameters of the molecule.^42^ For efficient energy conversion, the HOMO value of the molecule must be less than the HOMO value of the redox couple I_3_^–^/I, i.e., −4.9 eV,^43^ while the value of LUMO of the dye molecule must be greater than the value of CB of the semiconductor TiO_2_, i.e., −4 eV.^44^ Different functionals like BPV86, LSDA, and B3PW91 with basis set 6-311G in addition to B3LYP functionals with various basis sets 6-31G, 6-311G, and cc-PVDZ were utilized for the optimizations and calculations, and comparative data are reported in Table 1. *E*_LUMO_, *E*_HOMO_ values of [**A10**–**A8(trans)**] dyes calculated with B3LYP functionals achieved *E*_HOMO_ from −5.29 to −6.08 eV and *E*_LUMO_ from −2.39 to −2.9 eV as shown in Table 2. These calculated values prove the ultrafast injection of electrons from the dye molecule into the CB of the TiO_2_ semiconductor. One leading dye molecule among other efficient dyes has achieved HOMO/LUMO at −5/–3.5 eV.^45^ This is a reasonable HOMO–LUMO gap, which is sufficient for expanding the region of energy conversion. The B3LYP calculated values of *E*_HOMO_ – *E*_LUMO_ gap (Δ*E*) of [**A1**–**A18(trans)**] dyes are given in Table 2.

**Table 2:** B3LYP (Otherwise Defined) Calculated Values of *E*_LUMO_, *E*_HOMO_, and *E*_HOMO_ – *E*_LUMO_ gap (Δ*E*) of **A1**–**A18** Dyes

dyes	*E*_LUMO_ (eV)	*E*_HOMO_ (eV)	*E*_HOMO_ – *E*_LUMO_ gap Δ*E* (eV)
**A1**	–2.84	–6.02	3.186
**A1(BPV86)**	–4.12	–5.33	1.21
**A1(LSDA)**	–4.73	–5.97	1.24
**A1(B3PW91)**	–3.27	–6.14	2.87
**A2**	–2.77	–6.08	3.302
**A2(BPV86)**	–3.83	–5.30	1.47
**A2(LSDA)**	–4.48	–5.83	1.35
A2(B3PW91)	–2.98	–6.13	3.15
**A3**	–2.62	–5.93	3.316
**A3(BPV86)**	–3.75	–5.20	1.45
**A3(LSDA)**	–4.34	–5.71	1.37
**A3(B3PW91)**	–2.88	–6.02	3.14
**A4**	–2.39	–5.29	2.897
**A5**	–2.49	–5.42	2.93
**A6**	–2.49	–5.34	2.85
**A7**	–2.61	–5.66	3.06
**A8**	–2.58	–5.39	2.81
**A9(cis)**	–2.75	–5.82	3.07
**A9**_**DMSO**_**(cis)**	–2.79	–5.79	3.01
**A9(trans)**	–2.9	–5.71	2.83
**A10**	–2.6	–5.58	2.98
**A11**	–2.52	–5.12	2.6
**A12**	–2.54	–5.36	2.82
**A13**	–2.73	–5.64	2.92
**A14**	–2.69	–5.75	3.05
**A15**	–2.62	–5.73	3.1
**A16(cis)**	–2.44	–5.58	3.14
**A16(trans)**	–2.53	–5.48	2.95
**A17**	–2.75	–5.75	2.99
**A18**	–2.74	–5.77	3.02

### 3.2 Reactivity Descriptors

The parameters of the chemical reactivity of the dye molecule show the ability of molecule to stabilize in its environment.^46^ Different functionals like BPV86, LSDA, and B3PW91 with the basis set 6-311G in addition to B3LYP functionals with various basis sets 6-31G, 6-311G, and cc-PVDZ were utilized for the calculations and comparative data reported in Table 3. Different chemical parameters, which were calculated and reported in Table 3 such as the chemical and ionization potential, the global hardness and softness, the DM, and the transition dipole moment (TDM) well explained the chemical attitude of dye molecules. The HOMO–LUMO energy gap largely affected the hardness and softness of the molecule, that is, a lower energy gap is associated with the softness of the molecule and a higher energy gap value indicates the hardness of the molecule.^47^ Similarly, ionization potential (IP) values are responsible for the reactivity parameters of the molecule as a greater value of IP increases the reactivity of the molecule. Moreover, less softness value and high hardness value are responsible for the high stability and less reactivity of the dye as given in Table 3. **A1**, **A2**, **A3**, and **A15** are found to be the most stable thermally and kinetically among all studied dye molecules because of calculated global hardness and chemical potential values. From the negative potential value, we observed that the dyes have a greater ability to gain electrons from the environment. So, most of the dyes efficiently convert solar energy to electrical energy due to their high reactivity and high charge- transfer tendency.

**Table 3:** DFT-B3LYP (Otherwise Defined) Calculated Chemical Reactivity Parameters of **A1**–**A18** Dyes

dyes	electron affinity (EA)	ionization potential (IP)	electronegativity (*x*)	electrophilicity (ω)	chemical potential (μ)	global hardness (η)	global softness (σ)	DM (D)
**A1**	2.84	6.02	4.43	0.359	–1.59	4.429	0.113	5.605
**A1(BPV86)**	4.12	5.33	5.03	0.035	–0.60	5.03	0.099	5.967
**A1(LSDA)**	4.73	5.97	5.04	0.038	–0.62	5.04	0.099	6.066
**A1(B3PW91)**	3.27	6.14	4.70	0.304	–1.43	4.70	0.106	5.868
**A2**	2.77	6.08	4.425	0.386	–1.65	4.275	0.117	5.967
**A2(BPV86)**	3.83	5.30	4.56	0.160	–0.73	4.56	0.109	5.884
**A2(LSDA)**	4.48	5.83	5.15	0.130	–0.67	5.15	0.097	5.957
**A2(B3PW91)**	2.98	6.13	4.54	0.345	–1.57	4.54	0.110	5.785
**A3**	2.62	5.93	4.275	0.386	–1.65	4.275	0.117	2.755
**A3(BPV86)**	3.75	5.20	4.47	0.161	–0.72	4.47	0.111	2.745
**A3(LSDA)**	4.34	5.71	5.02	0.135	–0.68	5.02	0.099	2.782
**A3(B3PW91)**	2.88	6.02	4.45	0.352	–1.57	4.45	0.112	2.755
**A4**	2.39	5.29	3.84	0.374	–1.45	3.873	0.129	1.321
**A5**	2.49	5.42	3.955	0.369	–1.46	3.95	0.126	1.47
**A6**	2.49	5.34	3.915	0.365	–1.43	3.916	0.128	1.472
**A7**	2.61	5.66	4.135	0.370	–1.53	4.134	0.121	5.668
**A8**	2.58	5.39	3.985	0.354	–1.41	3.983	0.126	2.649
**A9(cis)**	2.75	5.82	4.285	0.357	–1.53	4.286	0.117	6.886
**A9**_**DMSO**_**(cis)**	2.79	5.79	4.29	0.316	–1.50	4.22	0.102	6.79
**A9(trans)**	2.9	5.71	4.305	0.328	–1.41	4.3	0.116	8.98
**A10**	2.6	5.58	4.09	0.364	–1.49	4.088	0.122	2.636
**A11**	2.52	5.12	3.82	0.340	–1.3	3.82	0.131	1.89
**A12**	2.54	5.36	3.95	0.356	–1.41	3.95	0.127	2.174
**A13**	2.73	5.64	4.185	0.348	–1.46	4.19	0.119	6.33
**A14**	2.69	5.75	4.22	0.360	–1.52	4.22	0.118	6.734
**A15**	2.62	5.73	4.175	0.371	–1.55	4.17	0.119	7.64
**A16(cis)**	2.44	5.58	4.01	0.391	–1.57	4.01	0.125	7.5
**A16(trans)**	2.53	5.48	4.005	0.368	–1.47	4.0	0.125	10.26
**A17**	2.75	5.75	4.25	0.351	–1.49	4.25	0.118	4.26
**A18**	2.74	5.77	4.255	0.171	–1.51	8.84	0.057	7.5

### 3.3 Spectral Properties

Although dye molecules show more sensitivity toward the absorption spectrum in the visible region, conventional dyes are sensitive in the visible region of the electromagnetic spectrum. Photons of wavelength less than 920 nm can be used in the photovoltaic conversion of universal AM 1.5 illumination into electricity.^48^ In DSSCs, UV light can be useful by using materials with low conversion luminescence.^49^ The DFT spectral studies of dye molecules in the UV region predict the degradation of dye upon UV exposure.^50^ Therefore, the most competent and leading organic dye is the one that shows a strong excitation peak in the visible spectral region, which is very helpful in understanding the prospective transition of electrons from the dye to the semiconductor surface.

**Figure 3:**
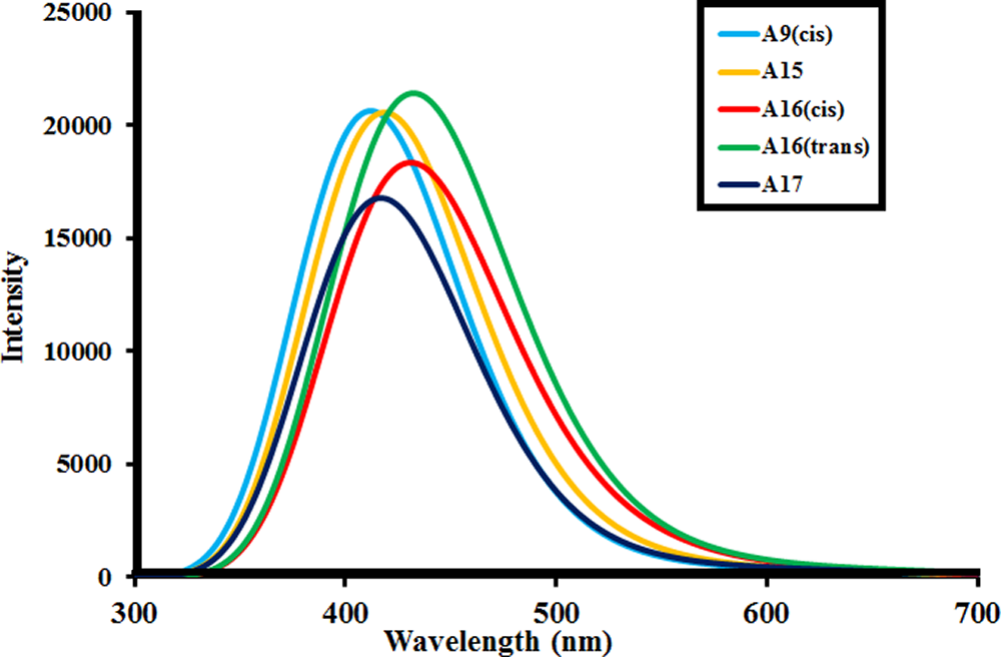
TD-DFT-B3LYP calculated excitation spectra of representative guanidine-based dyes [**A9(cis)**, **A15**, **A16(cis)**, **A16(trans)**, and **A17**].

**Figure 4:**
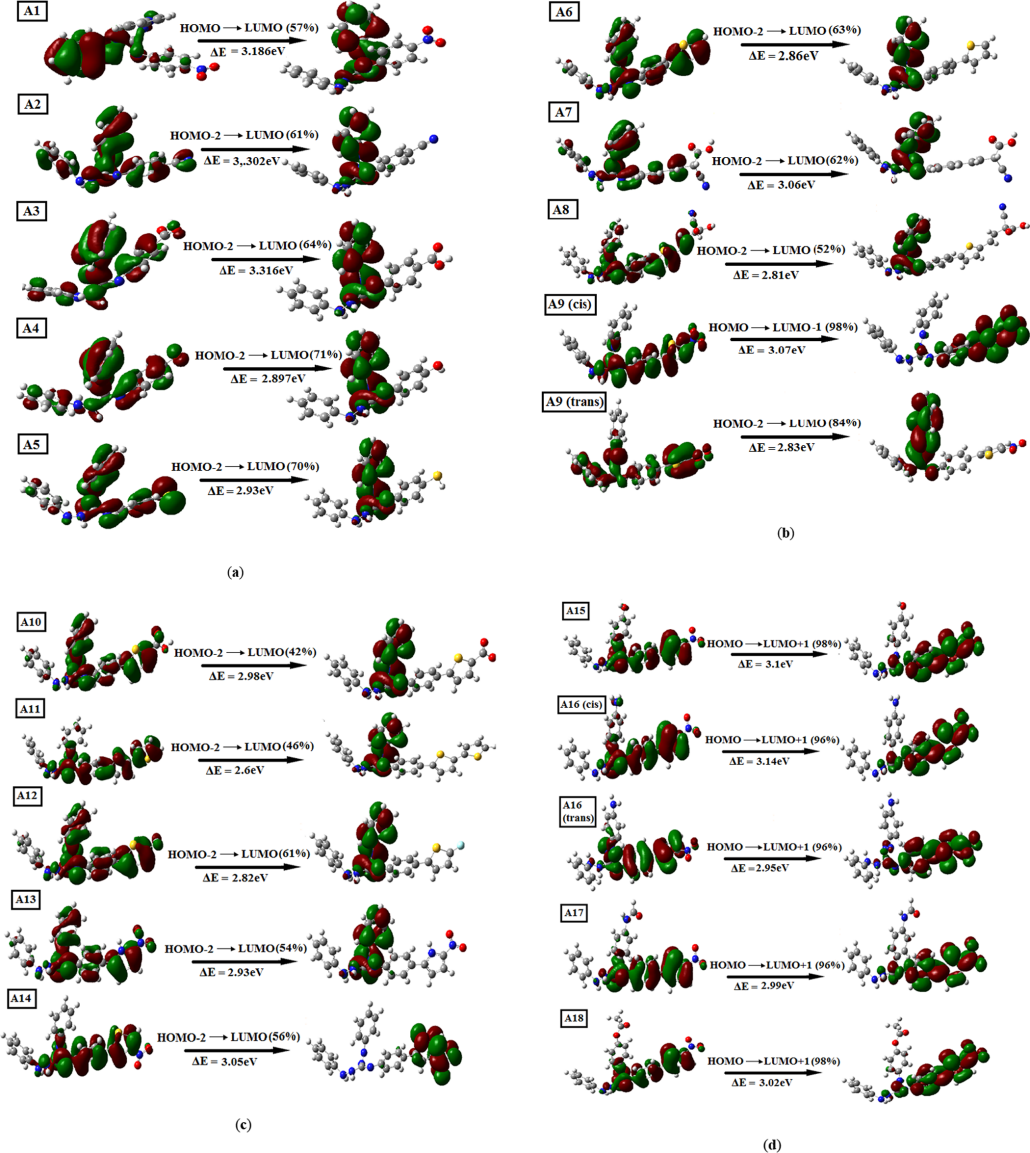
(a) FMOs for main transitions of guanidine-based dyes (**A1**–**A5)** using the DFT-B3LYP method, (b) FMOs for main transitions of guanidine-based dyes [**A6**–**A9(trans)**] using the DFT-B3LYP method, (c) FMOs for main transitions of guanidine-based dyes (**A10**–**A14**) using the DFT-B3LYP method, and (d) FMOs for main transitions of guanidine-based dyes (**A15**–**A18**) using the DFT-B3LYP method.

Elucidation of spectral properties of dyes was done through TD-DFT-B3LYP at the ground-state equilibrium geometry (enlisted in Table 4), and conclusive simulated excitation spectra are shown in Figure 3. The transition of intramolecular charge transfer (ICT) is directly affected by the light LHE. **A1**–**A18** dyes have shown more than 40% transfer of charge from the corresponding HOMO levels to the respective LUMO levels as shown in Figure 4a–d. **A9(cis)**, **A15, A16(cis)**, **A16(trans)**, **A17**, and **A18** dyes have exhibited more than 95% charge transfer from H-0 → L + 1 transition as shown in Table 4. It has been studied that the most effective energy gap (Δ*E*) should be in the range between 1.1 and 2 eV to get an efficient value of LHE.^51^ Energy gap (Δ*E*) for [**A1**–**A18(trans)**] dyes was found to be greater than 2.0 eV. Oscillator strength describes the strength of absorbing energy in the solar spectrum. LHE increases with the increasing value of oscillator strength as LHE = 1^–10^*f* where *f* is the oscillator strength.^52^**A9(cis)**, **A15, A16(cis)**, **A16(trans)**, **A17,** and **A18** dyes were found to have higher oscillator strengths and hence significant LHEs as depicted in Table 4. TDM is also related to the oscillator strength. All [**A1**–**A18(trans)**] dyes showed that the structural axis of the dye is responsible for the TDM. In the study of all dye molecules, **A9 (cis)** and **A9(trans)** exhibited the highest value of TDM as well as a high value of oscillator strength.

**Table 4:** TD-DFT-B3LYP Calculated Spectral Parameters of **A1**–**A18** Dyes

dyes	λ_max_	*E*_HOMO_ – *E*_LUMO_ gap (Δ*E*) (eV)	oscillator strength *f* (a.u)	TDM (a.u)	major transitions	LHE
**A1**	476.8	3.186	0.02	0.3143	H-0→L+0 (57%)	0.05
**A2**	385	3.302	0.012	0.1564	H-2→L+0 (61%)	0.03
**A3**	382.37	3.316	0.013	0.168	H-2→L+0 (64%)	0.03
**A4**	399.96	2.897	0.013	0.1728	H-2→L+0 (71%)	0.03
**A5**	405.93	2.93	0.013	0.1712	H-2→L+0 (70%)	0.03
**A6**	412.57	2.85	0.011	0.1516	H-2→L+0 (63%)	0.02
**A7**	397.72	3.06	0.013	0.1687	H-2→L+0 (62%)	0.03
**A8**	421	2.81	0.0083	0.1158	H-2→L+0 (52%)	0.02
**A9(cis)**	412	3.07	0.509	0.69	H-0→L+1 (98%)	0.69
**A9**_**DMSO**_**(cis)**	412	3.01	0.501	0.67	H-0→L+1 (98%)	0.67
**A9(trans)**	432.76	2.83	0.048	0.68	H-2→L+1 (84%)	0.11
**A10**	406	2.98	0.011	0.1451	H-2→L+0 (44%)	0.02
**A11**	633	2.6	0.0064	0.1329	H-2→L+0 (46%)	0.02
**A12**	416	2.82	0.01	0.1451	H-2→L+0 (61%)	0.02
**A13**	413	2.92	0.009	0.1245	H-2→L+0 (54%)	0.02
**A14**	402	3.05	0.01	0.1286	H-0→L+1 (56%)	0.02
**A15**	418	3.1	0.506	6.98	H-0→L+1 (98%)	0.69
**A16(cis)**	430	3.14	0.449	6.37	H-0→L+1 (96%)	0.65
**A16(trans)**	432	2.95	0.523	7.44	H-0→L+1 (96%)	0.7
**A17**	416	2.99	0.414	5.68	H-0→L+1 (97%)	0.61
**A18**	414	3.02	0.464	6.34	H-0→L+1 (98%)	0.66

TD-DFT-B3LYP-calculated excitation spectra of representative guanine-derived dyes [**A9(cis)**, **A15**, **A16(cis)**, **A16(trans)**, and **A17**] are presented in Figure 3. It has been analyzed from wave functions that absorption (λ_max_) is due to the transition of electrons from the ground-state HOMO level to the excited-state LUMO level. The excitation peaks describe the values of LHE which in turn is related to the oscillator strength values (given in Table 4), and molecular orbitals are involved in the major transition of electrons, as mentioned in Figure 4a–d. It was observed that the change in the basic guanidine subunit by introducing thiophene substituted with a specific functional group remarkably effected the π-conjugation that may be responsible for the HOMO–LUMO gap as well as upshift and downshift of the absorption region of the spectrum. These absorption maxima in the 300–600 nm range correspond to the maximum oscillator strengths for each dye molecule. The highest intensity peak was observed for [**A9(cis)**, **A15**, **A16(cis)**, **A16(trans),** and **A17**] which have a nitro group-substituted thiophene ring that is attached to the phenyl ring of the guanidine subunit. As thiophene units are reported, there is a consequent increase in the length of the D-π-A type dye, which causes a redshift of the absorption bands toward the visible region.^13^**A9(cis)** has shown that maximum absorption at 411 nm corresponds to charge transfer (98%) between major molecular orbitals involved (H-0→L+1). **A15** shows that the maximum absorption at 420 nm corresponds to charge transfer (98%) between major molecular orbitals involved (H-0→L+1). **A16(cis)** and **A16(trans)** show maximum absorption at 432 nm, which corresponds to charge transfer (96%) between major molecular orbitals involved (H-0→L+1). **A17** shows maximum absorption at 417 nm, which corresponds to charge transfer (97%) between the major molecular orbitals involved (H-0→L+1).

### 3.4 DFT Study of Dye-TiO_2_ Interaction

**Figure 5:**
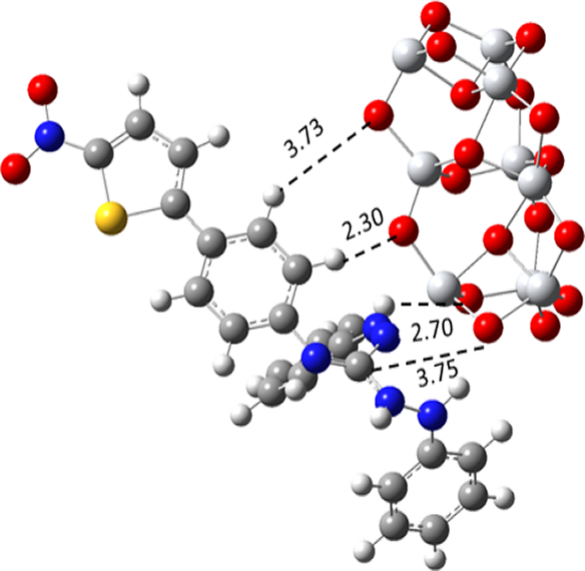
Optimized geometric structure of the dye A9(cis)-Ti_9_O_18_ bounded complex.

**Figure 6:**
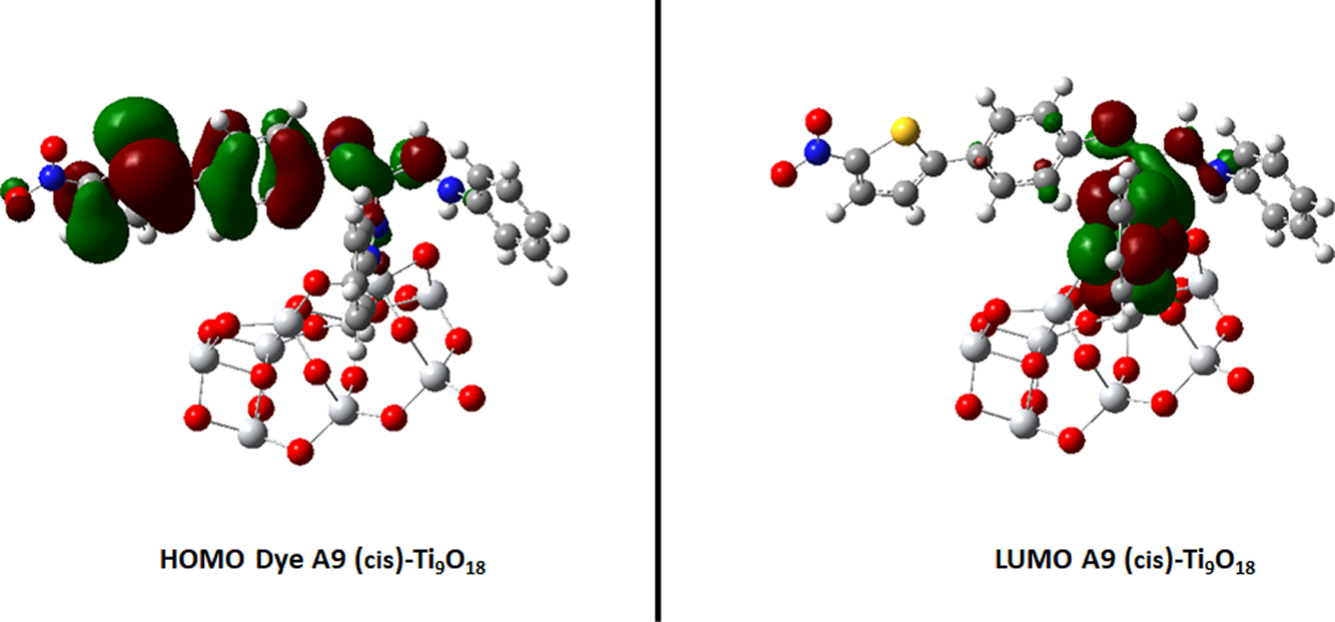
FMOs of the dye A9(cis)-Ti_9_O_18_ bounded complex.

TiO_2_, as a nanoparticle, is a photoactive material because of its stability. It has potential applications in solar energy conversion and keeping the environment green. DFT study of organic dyes with the interaction with TiO_2_ prior to synthesizing the organic dye as the photosensitizer is very important to check its efficiency toward DSSCs. For the DFT study, the dye-TiO_2_ interaction was carried out at Gaussian 16W using the B3LYP hybrid functional and LanL2DZ basis set. First, TiO_2_ oligomers with up to nine repeating units were optimized, followed by the calculation of their interaction with the dye. There are different intermolecular interactions observed between the C and H of the dye with the O atom of TiO_2_, as mentioned in Figure 5. Band gap of dye A9-cis is 3.07 eV and band gap of TiO_2_ is 2.05 while on interaction, and the band gap between HOMO–LUMO became 1.55 eV as mentioned in Figure 6. This decrease in band gap ensures the excellent photoactive property of the dye which is correlated with the π-conjugation of the organic dye.

### 3.5 Molecular Electrostatic Potential Reactivity Study

**Figure 7:**
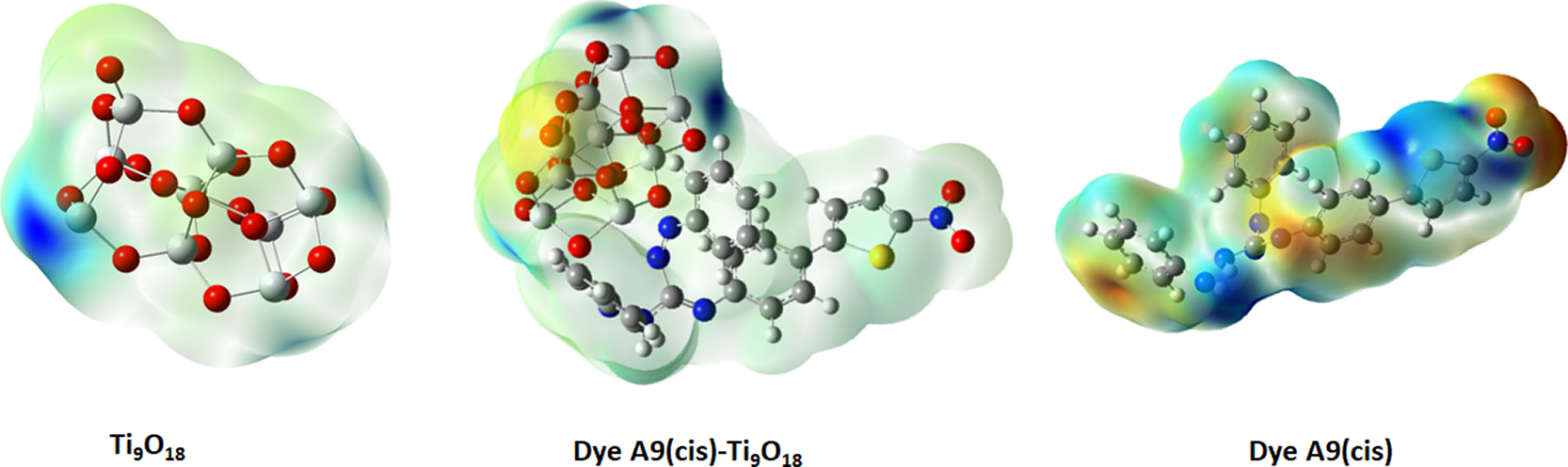
MEP plots of Ti_9_O_18_, dye A9(cis)-Ti_9_O_18_, and dye A9(cis).

Molecular electrostatic potential (MEP) was carried out by using Gaussian 16W/B3LYP/LanL2DZ in the gas phase with TD-DFT as shown in Figure 7. For A9 (cis) compound contour plots, the optimized structure and MEP surfaces were evaluated, and nucleophilic and electrophilic properties were explained using different colors. Red indicates higher energy area and explains the electrophilic (attractive) potential, and blue indicates lower energy area which explains the nucleophilic (repulsive) potential. Electrostatic potential reduces in the order as red < orange < yellow < green < blue.

### 3.6 Vibrational Analysis

**Figure 8:**
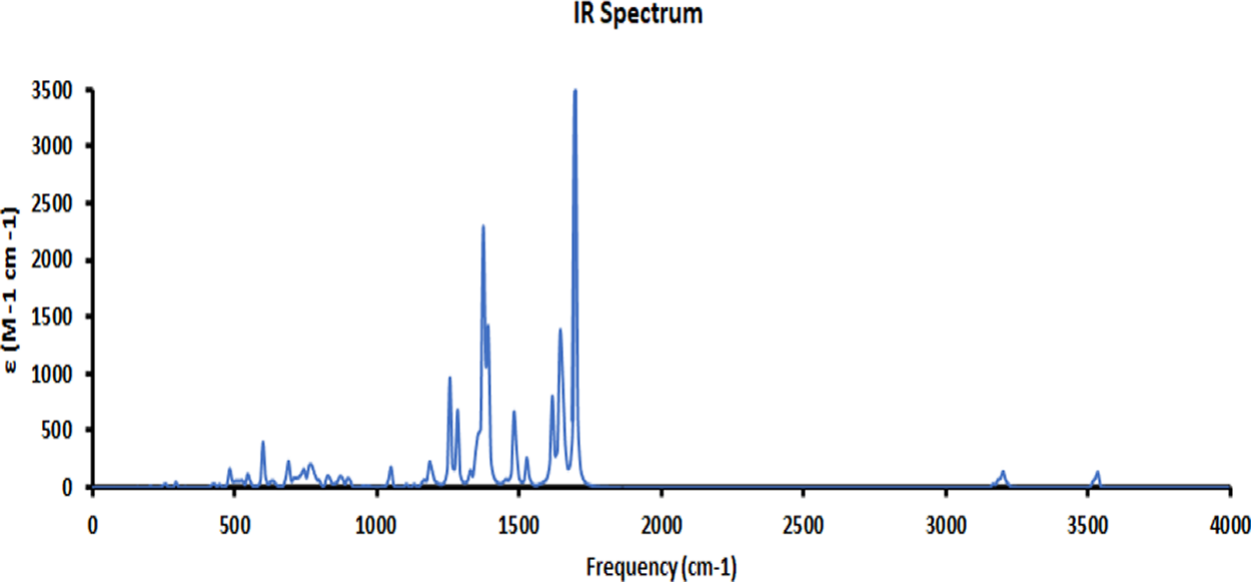
IR spectrum of dye A9(cis).

Theoretical vibrational analysis was calculated by using Gaussian 16W under hybrid functional B3LYP with the LanL2DZ basis set, as shown in Figure 8. It was observed from the spectrum obtained by calculating the frequency of the A9 (cis) molecule that strong intensity of absorption around 3200–3400 cm^–1^ indicates the presence of the N–H bond. Aromatic C–H and C–C bonds express their peaks in the normal region. Azo linkage in the compound was indicated in the range 1400–1520 cm^–1^, and the guanidine center was observed in the band range 1550–1700 cm^–1^.

### 3.7 Conformational Analysis

**Figure 9:**
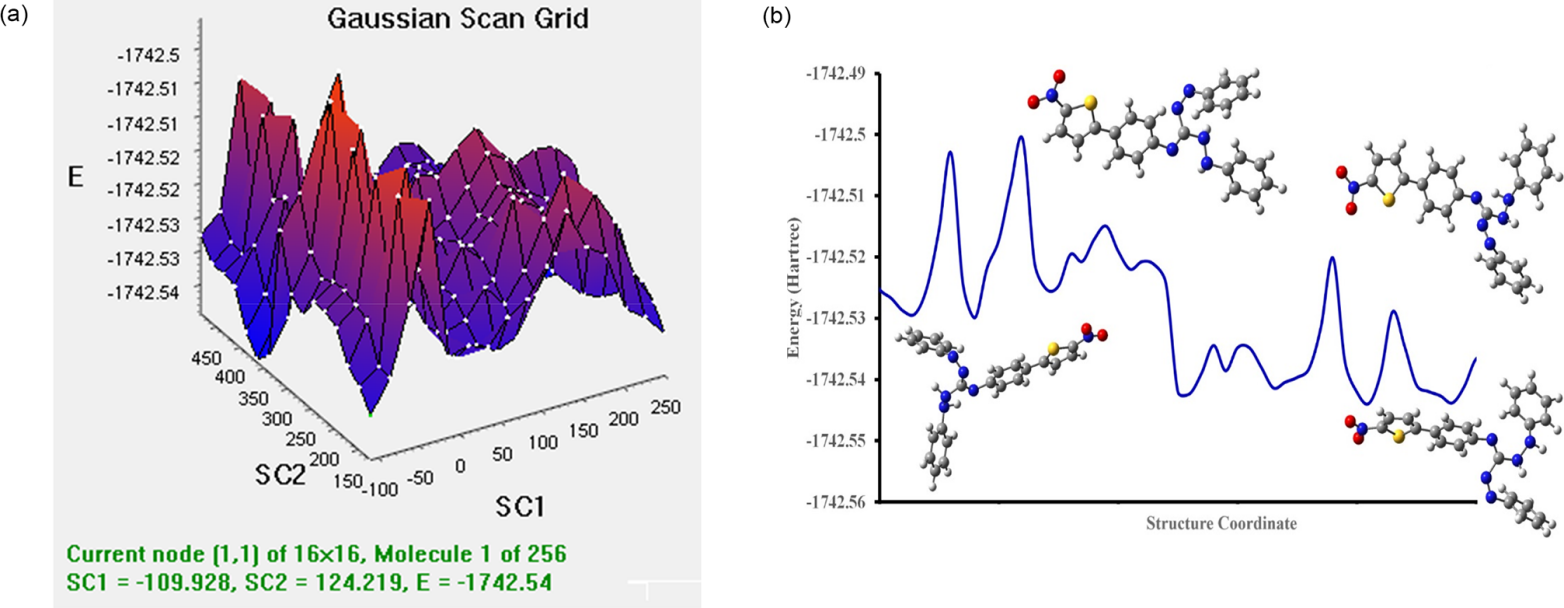
(a) PES of dye A9(cis). (b) PES grid of dye A9(cis).

Conformational analysis was performed using DFT/B3LYP/LanL2DZ for A9(cis). Conformers of A9(cis) with minima were evaluated using potential energy scan (PES) as shown in Figure 9a. It indicates four conformers with minima with total energy and scan coordinates of −1742.51574, −1742.50824, −1742.50824, and −1742.51574 kcal/mol. DFT scans suggest a total energy of −1742.54 kcal/mol with the scan coordinate 1 at −109.928 and scan coordinate 2 at 124.219 as shown in Figure 9b.

## 4 Conclusions

We explored the optical and electronic attitudes of guanidine-based organic dye molecules (**A1**–**A18**) computationally by using DFT under the B3LYP functional with various basis sets. The geometry of dye molecules in the ground state was completely optimized to study the properties of FMOs along with the excitation spectra in detail. The calculated values obtained from the computational studies of guanidine-based dyes showed that the optical properties such as energy gaps, excitation properties, TDM, oscillator strength, and LHE depend on the structural modifications of the dyes. It has been found that dyes with a thiophene moiety substituted with a NO_2_ group have good light LHEs. The density of charge was more dependent on the inner levels of HOMO. Therefore, ICT from inner HOMO levels is responsible for increasing excitation energy. The chromophore groups present in guanidine-based dyes are responsible to give absorption spectra in the visible region. Better anchorage of dye molecules to the surface of TiO_2_ semiconductors helps in charge-transfer phenomena, and the results suggested that −COOH, – CN, and–NO_2_ proved as proficient anchoring groups making dyes very beneficial and effective candidates for DSSCs. The high negative value of HOMO levels as compared to the redox couple (I^–^/I^3–^) reduction proves that these dyes are promising and appropriate leading molecules for superfast transfer of electrons into the CB of TiO_2_. Similarly, various other parameters, such as MEP, vibrational analysis, and conformational analysis, explained the high efficiency of organic dyes in solar cell fabrication. The high oscillator strength and good excitation spectra of these dyes give greater LHE, which increases the suitability of dyes for application in DSSCs.

## Data Availability

There is no associated data with this article.
